# Neural Modulation in Aversive Emotion Processing: An Independent Component Analysis Study

**DOI:** 10.1155/2016/2816567

**Published:** 2016-08-08

**Authors:** César Romero-Rebollar, Luis Jiménez-Ángeles, Eduardo Antonio Dragustinovis-Ruiz, Verónica Medina-Bañuelos

**Affiliations:** ^1^Neuroimaging Laboratory, Department of Electrical Engineering, Universidad Autónoma Metropolitana, 09340 Mexico City, Mexico; ^2^Department of Biomedical Systems, Faculty of Engineering, Universidad Nacional Autónoma de México, 04510 Mexico City, Mexico

## Abstract

Emotional processing has an important role in social interaction. We report the findings about the Independent Component Analysis carried out on a fMRI set obtained with a paradigm of face emotional processing. The results showed that an independent component, mainly cerebellar-medial-frontal, had a positive modulation associated with fear processing. Also, another independent component, mainly parahippocampal-prefrontal, showed a negative modulation that could be associated with implicit reappraisal of emotional stimuli. Independent Component Analysis could serve as a method to understand complex cognitive processes and their underlying neural dynamics.

## 1. Introduction

Emotion processing is crucial for social interaction. It has been suggested that both neuronal and behavioral responses are facilitated, whenever emotional faces with negative valence, such as fear or anger, are observed. It has been reported that the perception of affective scary faces indicates the presence of indirect stimuli that could potentially threaten individual integrity [1].

The processing of affective faces of fear has an important social role, because it triggers emotional information capable of provoking a visceral response and thus enabling the necessary actions to preserve physical integrity [2]. It also allows the detection of other persons' emotional state to modulate our responses during social interaction, by controlling or attenuating conduct; this is due to the human ability to consciously assess emotional stimuli through continuous reasoning and experience labeling [2–4].

A brain circuit implicated in emotions' processing has been described; it is composed mainly of the amygdala, anterior cingulate, insula, prefrontal cortex (mainly ventromedial and lateral orbitofrontal portions), and anterior portions of the temporal lobe [5–7].

Several paradigms have been used in fMRI studies to assess affective faces processing. Experiments have been reported, going from passive perception of emotional faces to those including conditions where the subject must produce a behavioral response involving decision making; in this case, responses can be either implicit, that is, the selection or judgment about the identity or gender of stimuli, or explicit, where emotions are assessed directly. Consistent findings have been reported in healthy and clinical populations, describing the increase in the BOLD response when aversive affective faces are compared to neutral faces, mainly in the amygdala; frontal regions such as the superior frontal gyrus, medial frontal gyrus, orbitofrontal cortex, and ventromedial cortex; temporal regions including the superior and medial temporal gyrus; other regions such as the fusiform gyrus, insula, anterior cingulate, among other structures [8–11].

Processing of aversive faces is also considered a biomarker of negative affectivity, which has been associated with neuropsychiatric pathologies and maladaptive personality traits. In a recent study, it is reported that an affective-cognitive bias (or the tendency to respond faster to a stimulus) facilitated the recognition of fear faces and led to shorter reaction times (compared to neutral faces), which correlated positively with a bilateral increment of ventromedial cortex, left subgenual cortex, and right caudate nucleus activities. This bias was also associated with personality traits such as harm avoidance [1]. In another study, carried out in a population of heroin-dependent subjects, an increase in the left amygdala activity was observed, when heroin was administered to participants during the fearful face condition; this increase was positively correlated with other measures related to stress [12].

Face Matching Task (FMT) [2, 13] is one of the most commonly used paradigms to study affective processing; different versions have been adapted, which consider several conditions, but all of them allow the implicit and explicit assessment of affective processing, including aversive emotions, such as fear or anger (see Materials and Methods for the task description). In several studies, mainly based on region of interest (ROI) analysis and small volume correction analysis (SVC), the amygdala has been associated with FMT resolution, especially in aversive conditions [2, 13]; furthermore, other hyperactive regions besides the amygdala have been observed, such as ventromedial cortex, orbitofrontal cortex, insula, and anterior cingulate in diverse populations of patients with affective disorders, for instance, acute stress [14], depressive episodes [15–17], and first psychotic episode [18]. These findings suggest that the performance during FMT can be considered a biological marker of emotional reactivity to aversive stimuli, besides being a consistent endophenotype of genetic susceptibility to the development of affective disorders characterized by emotional hypersensibility [19–21] and of traits such as impulsivity, aggression, and violence [22].

The BOLD signal obtained by fMRI is based on the general linear model (GLM); its traditional analysis presents some limitations: on the one hand, it assumes voxels' independency, which limits the BOLD signal study to massive univariate analysis, which in most cases cannot capture the principle of biological plausibility, namely, the biological mechanism that underlies a process, such as cognitive functioning; on the other hand, it needs a reference model based on the hemodynamic response, which makes the analysis based on GLM less flexible than other approaches [23].

An alternative to study and to characterize the neural networks associated with cognitive processing is functional connectivity; for its study several techniques, generally expressing the statistical dependency on observed data, have been proposed. Among them, Independent Component Analysis (ICA) is a multivariate technique that allows the observed BOLD signals' decomposition into neural networks or independent components (ICs). ICs refer to functionally independent neural networks that are simultaneously activated [24]. After elimination of noise-related signals and the proper selection, these ICs represent the activity modulation of a set of brain structures that, in the case of fMRI, are time-related either to stimulus presentation or to a given condition. This technique does not depend on a reference model and as a final result allows the analysis of components, instead of voxels; that is, the brain regions represented by each component have a similar response associated with the cognitive process of interest and show temporarily coherent fluctuations [23].

There are only a few studies, where ICA has been applied to observe the neural networks modulation during emotion processing. Escartí et al. [25] used an auditory paradigm, where they manipulated the words' emotional tone; for a control group they reported four ICs temporally correlated with emotional stimuli, located at temporal, frontoparietotemporal, limbic-subcortical, and occipitocerebelar regions. The limbic-subcortical IC was then compared between schizophrenic subjects with and without auditory hallucinations, and they found a similar behavior between control and nonhallucinating subjects, while schizophrenic patients with hallucinations presented a hyperactivation of this component.

In another ICA-based study a group of women with borderline personality was compared to a control group. A paradigm including neutral, masked fear and explicit fearful faces was used; the responses' analysis yielded a bilateral component that included the amygdala as a “seed” coactivated with the rostral portion of the anterior cingulate in the explicit fear condition; this functional connectivity was increased for the borderline personality group [26].

Broicher et al. [27] reported an amygdala IC, which showed coactivations with temporal, frontal, anterior cingulate, and hippocampal and cerebellar regions, under the passive view of intense fear faces videos; the functional connectivity of the amygdala with the aforementioned regions was reduced in a group of temporal lobe epilepsy patients.

ICA has also helped to describe active networks in basal or resting state conditions; it has been suggested that those networks have a strong contribution for the development of some pathologies. In a study carried out in raped female teenagers, compared to a control group, fearful faces stimuli were used to observe the activity modulation of three networks associated with resting state: frontal-parietal, frontal-cingulate, and default mode network (DMN). The authors reported that the frontal-cingulate network, composed mainly of the anterior insula and anterior cingulate, showed an increased activity in the fear condition, more so in women having suffered a rape event [28]. Another study in the same direction reported that the structures overlapping DMN (prefrontal medial cortex, ventral anterior cingulate, and precuneus) showed a negative modulation or deactivation to emotional stimuli, which suggests that DMN participates in the monitoring of internal emotional processing [29].

To our knowledge, only one study of functional connectivity using the FMT has been reported. In that work, the time courses corresponding to the amygdala were extracted and correlation maps were computed between these time courses and those corresponding to all the other voxels, with the purpose of describing how the amygdala's activity modulates the rest of the regions when processing aversive emotions. It was reported that the right amygdala activation had a negative modulation on medial and superior frontal regions, anterior cingulate, inferior parietal, precuneus, and cuneus. On the other hand, the right amygdala activity modulated positively the activities of inferior frontal gyrus, insula, and superior temporal and subcortical regions [30].

In summary, the findings about the brain regions implicated in emotional processing have been consistent in the literature. However, few studies have been reported using an approximation of functional connectivity, such as ICA, that captures the principles of biological plausibility, fundamental to understand cognitive processing; that is, it assesses the positive and negative modulations of temporally coherent neural networks. Furthermore, ICA have not been used to analyze responses to the widely used FMT paradigm, which has been suggested as a good biological marker of emotional reactivity and as a reliable endophenotype of genetic susceptibility to the development of affective disorders and maladaptive behaviors. Therefore, the purpose of the present study was to characterize the neural networks implicated in aversive emotional processing measured by FMT, using Independent Component Analysis. We hypothesized that ICs temporally associated with fearful stimuli processing will correspond to brain regions implicated in perception and regulation of aversive emotion stimuli, as well as negative affectivity, such as prefrontal, ventromedial, orbitofrontal, and anterior temporal areas.

## 2. Materials and Methods

### 2.1. Participants

The sample was composed of ten healthy adults (5 males, 5 females) with a mean age of 25 ± 5.29 years and a mean of 15.5 ± 2.32 years of education (see Table S1 of the Supplementary Material for more details about sample selection available online at http://dx.doi.org/10.1155/2016/2816567). All subjects signed an informed consent; they did not receive economical compensation for their participation; the project was approved by the ethics committee of the Centro Nacional de Investigación en Imagenología e Instrumentación Médica of the Universidad Autónoma Metropolitana.

### 2.2. fMRI Paradigm

**Figure 1:**
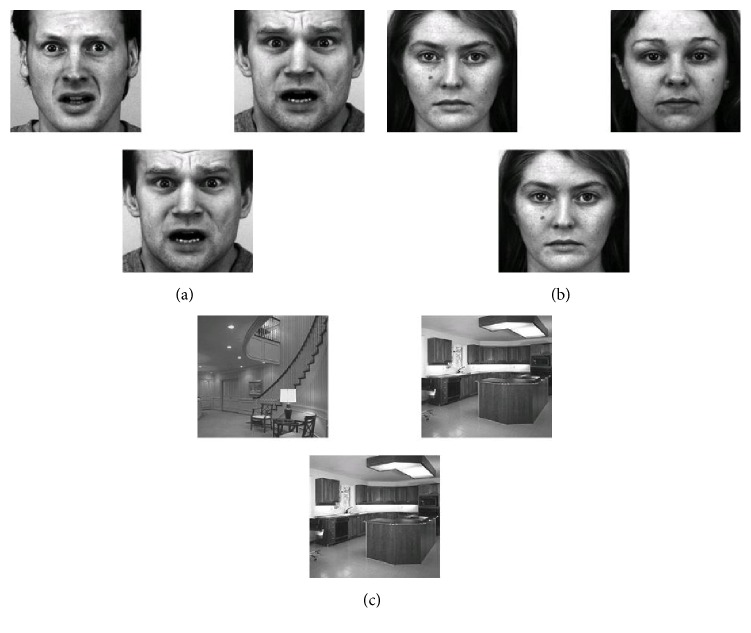
FMT conditions. (a) Fear. (b) Neutral. (c) Control.

An adaptation of Face Matching Task (FMT) [2, 13] was developed; in this perceptual task subjects saw a trio of faces and they have to select one of the two faces (top) that was identical to the face in the bottom of the screen, so it was an implicit emotional task in which the subject made a judgment about the identity of the stimulus. Trials were presented in an event-related design. A total of 48 emotional faces (24 neutral, 24 fear) derived from a set of affective emotional faces were presented [31]. In addition, 24 sensory-motor control stimuli, in which the emotional faces were replaced by scenes of interiors of houses, were presented in an interleaved manner with emotional faces. Each trial was presented sequentially in a pseudorandom order during 2000 ms with an interstimulus interval of 2100 ms. In summary, the fMRI paradigm consisted of three conditions: aversive affect processing (fear), neutral affective processing (neutral), and sensory-motor control (control) (Figure 1). The experimental paradigm was presented by E-Prime 2.0 software (Psychology Software Tools, Pittsburg, PA, USA); stimuli were projected in a BOLD-screen (Cambridge Research Systems); reaction times (RT) were recorded using a two-button response pad (Current Designs).

### 2.3. Image Acquisition

Structural and functional magnetic resonance images were acquired in a Philips 3T Achieva scanner (Philips Medical Systems) using an 8-channel SENSE Head coil. Functional images were acquired using a Gradient-Echo Planar Imaging (EPI) sequence with the following parameters: TR = 2000 ms; TE = 28 ms; acquisition matrix = 80 × 80; voxel size = 1.87 mm × 1.87 mm × 5 mm; slice thickness = 4 mm; gap = 1 mm; flip angle = 90°; FOV = 128 × 128 mm; 24 axial slices, order of acquisition = interleaved. A 3D T1-weighted structural image was acquired for coregistration with the following parameters: TR = 7.5 ms; TE = 3.7 ms; acquisition matrix = 240 × 240; voxel size = 1 mm × 1 mm × 1 mm; slice thickness = 2 mm; no gap; flip angle = 8°; FOV = 256 × 256 mm.

### 2.4. Image Preprocessing

Images were preprocessed using Statistical Parametric Mapping software (SPM12, http://www.fil.ion.ucl.ac.uk/spm/) implemented in Matlab 2014b (Math Works, Natick, MA, USA). Functional images were realigned to first volume, slice-timing-corrected, coregistered to structural image, normalized to MNI space with a voxel size of 2 × 2 × 2 mm^3^, and then smoothed with a Gaussian FWHM kernel of 8 mm.

### 2.5. GLM Analysis

First level analysis for each subject was carried out using SPM12; the three experimental conditions were included as regressors, applying the canonic hemodynamic response function without derivatives. The six motion-correction parameters of each subject were also included in the model. A high-pass filter with a cutoff point of 128 seconds was applied to time series. Statistical images of the following contrasts were obtained with *P* < 0.005 (uncorrected), *k* = 10: face processing activation (neutral + fear > control); fear activation (fear > neutral); fear activation controlled by sensory-motor activity (fear > neutral + control).

The second level whole-brain analysis was carried out through a one-sample *t*-test using the contrast images obtained in first level analysis, with *P* < 0.005 (uncorrected), *k* = 10. The labeling of coordinates was done according to the stereotactic atlas of Talairach and Tournoux [32], as implemented in Talairach Client tool [33, 34]. These results are presented in Table S2 and Figures S1, S2, and S3 of the Supplementary Material.

### 2.6. Independent Component Analysis (ICA)

ICA was carried out using the GIFT software (http://icatb.sourceforge.net/). The procedure to estimate independent components (ICs) consists of several steps: first, the optimal number of ICs is estimated following the minimum description length criteria (MDL) [35]. Then a two-step data reduction through principal component analysis is made [36]. ICs are then decomposed to obtain the final number of components previously estimated through MDL; in the present study this step was carried out using the Infomax algorithm [37]. An optional step is to test the stability of the estimated ICs via ICASSO [38]. Then spatial maps of ICs and the associated time courses are calculated using a back-reconstruction approach, considering the results from ICA and data reduction steps [39]. ICs are normalized to *z*-scores.

GIFT determines the brain structures associated with each time course using a random factor analysis (one-sample *t*-test) as implemented in SPM8 with a threshold of *P* < 1 × 10^−12^ FDR-corrected; *k* = 30. This procedure allows obtaining spatial maps of the functionally connected brain structures in each IC.

In the present study 29 ICs were estimated via MDL; the Infomax algorithm was used and 20 iterations were carried out using ICASSO method. The number associated with each IC (IC1, IC2,…, IC29) is arbitrary and corresponds to the output of the GIFT platform.

The ICs of interest are usually selected as follows [40–42]:

(1) ICs with a stability index <0.9 in ICASSO must be removed; in the present study none of the estimated ICs were eliminated using this criterion.
(2) ICs are spatially sorted according to the templates of gray matter (GM), white matter (WM), and cerebrospinal fluid (CSF) included in SPM8; those ICs whose values of *R* ^2^ > 0.02 for WM, *R* ^2^ > 0.05 for CSF, and those whose value of *R* ^2^ for GM is lesser than that obtained for WM and/or CSF are also discarded. In this study, after performing this step 6 ICs with values of *R* ^2^ > 0.02 for WM were discarded; no ICs with *R* ^2^ > 0.05 for CSF were found; and 8 ICs with *R* ^2^ value for GM less than that obtained for the other tissues were discarded.
(3) The remaining 15 ICs were temporally sorted (multiple regression method) using the model estimation of the first level analysis, to obtain the beta values associated with the experimental conditions (control, neutral, and fear). The beta values represent the modulation of activity of the ICs temporally associated with the onset of the event convolved with the hemodynamic response. The modulation could be positive or negative, which corresponds, respectively, to activation or deactivation patterns during the stimulus processing.

### 2.7. Statistical Analysis

Reaction times and number of correct responses were analyzed using SPSS 20 software (SPSS, Chicago, IL). The number of correct responses was not normally distributed (*P*s < 0.05, Shapiro-Wilk) and therefore it was analyzed using a Friedman test for related samples. The pairwise comparisons were analyzed using the Wilcoxon signed-rank test for related samples, and a level of *P* < 0.05 was adopted. Reaction times were normally distributed (*P*s > 0.05, Shapiro-Wilk) and were analyzed using a repeated measures ANOVA; “condition” was included as within-subjects factor with three levels (control, neutral, and fear); a level of *P* < 0.05 was adopted.

The statistical analysis of ICs was carried out as follows: because the remaining 15 ICs were normally distributed (*P*s > 0.05, Shapiro-Wilk), a repeated-measures ANOVA for each IC was carried out, to detect those ICs that were differentially implicated in each condition, as within-subject factor “condition” was included with three levels (control, neutral, and fear). Afterwards another repeated-measures ANOVA for each IC was carried out, to detect the differences in the modulation between neutral and fear faces, as within-subjects factor “condition” was included with two levels. These analyses were carried out using SPSS 20; a level of significance of *P* ≤ 0.05 was adopted.

## 3. Results and Discussion

### 3.1. Behavioral Performance

The number of correct responses (CR) between conditions was statistically different (*P* = 0.001, Friedman) in all cases. In the control condition, subjects were more accurate (CR = 23.8 ± 0.42), followed by the fear condition (CR = 22.8 ± 0.63) and finally the neutral condition (CR = 21.5 ± 1.17). Pairwise comparisons indicated that all conditions were different (control versus neutral *P* = 0.005; control versus fear *P* = 0.020; neutral versus fear *P* = 0.023, all Wilcoxon).

A principal effect of “condition” on reaction time was found (*F*_2,18_ = 20,399, *P* < 0.000). The subjects responded faster in the control condition (reaction time = 967.4 ± 201.35 ms), followed by the fear condition (reaction time = 1057.2 ± 215.25 ms) and finally the neutral condition (reaction time = 1207.69 ± 297.92 ms). According to the results of pairwise comparisons, all conditions were different from each other.

Increases in accuracy and decreases in reaction times associated with fearful faces processing concur with previous reports indicating that the shift of attentional focus is manipulated during emotional processing in tasks similar to FMT [43]. The speed to process aversive emotional stimuli (fear) has been interpreted as a cognitive-affective bias to categorize fearful stimuli, even in healthy populations. It has been suggested that this bias is related to personality traits such as harming avoidance, which has been associated with no clinical traits of anxiety [1]. In the present study one of the inclusion criteria was to have normal levels of anxiety according to the Beck Anxiety Inventory; therefore, this speed in the categorization of fearful stimuli may be associated with a more cautious personality of our subjects, which does not imply a risk factor for the development of mood disorders [44].

### 3.2. ICA Results

**Figure 2:**
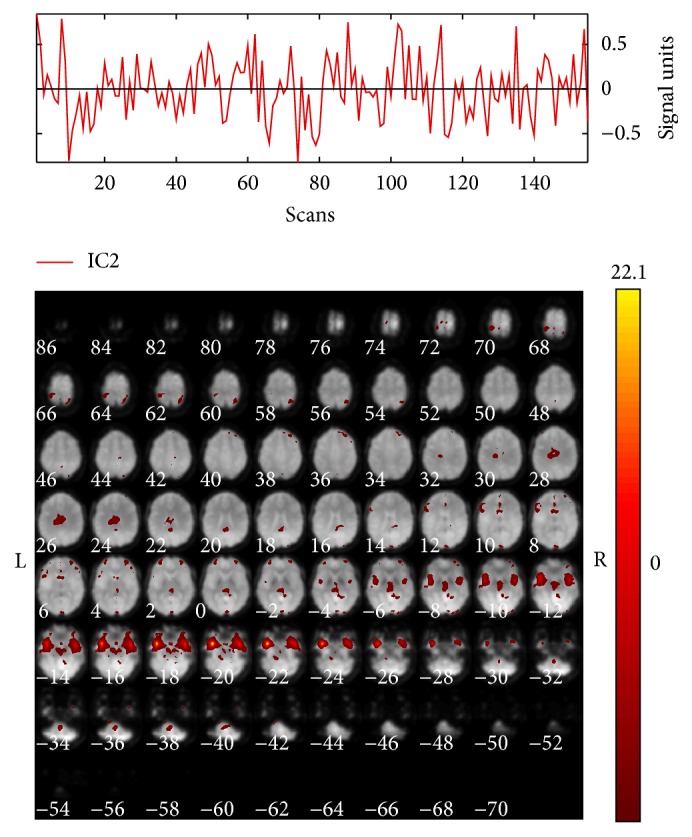
Spatial activation map and time course of IC2 “parahippocampal-prefrontal,” color bar represents *t* values.

**Figure 3:**
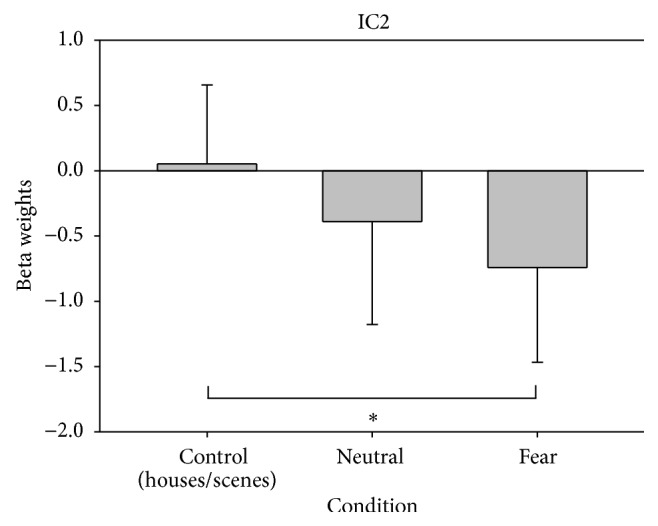
Modulation of IC2 in the three conditions of Face Matching Task. ^*∗*^ *P* < 0.05.

The comparison of the ICs in the 3 conditions (control, neutral, and fear) allowed detecting that modulation of IC2 was different between conditions (main effect of condition: *F*_2,18_ = 4.5, *P* = 0.025). Pairwise comparisons indicated that the difference was between control and fear conditions (*P* = 0.003); this component showed a negative modulation during the fear condition. The “Write Talairach Table” function implemented in GIFT with the default options was used to detect regions with a strong negative modulation within IC2. Due to the functionally connected brain regions, the IC2 was named as “parahippocampal-prefrontal” (Table 1, Figures 2 and 3).

This analysis, in which the sensory-motor control condition was included, allowed observing that the negative modulation of IC2 “parahippocampal-prefrontal” included structures involved in emotional face processing. It should be noted that in pairwise comparisons the fear condition was different from the control condition; however, the neutral condition also had a negative modulation, without being different from the other two conditions, suggesting that this negative modulation could be associated with the processing of emotional facial stimuli. It is worth noting that it has been reported that neutral faces processing shares the neural substrate of nonneutral emotional conditions, with the only difference that what defines the neuronal differential response in these brain structures is the sensitivity, depending on the emotional conditions [3, 4, 45]. It has also been reported by whole-brain analysis that neutral conditions are not different from those of fear [46], suggesting that neutral faces processing involves some emotional contribution.

**Figure 4:**
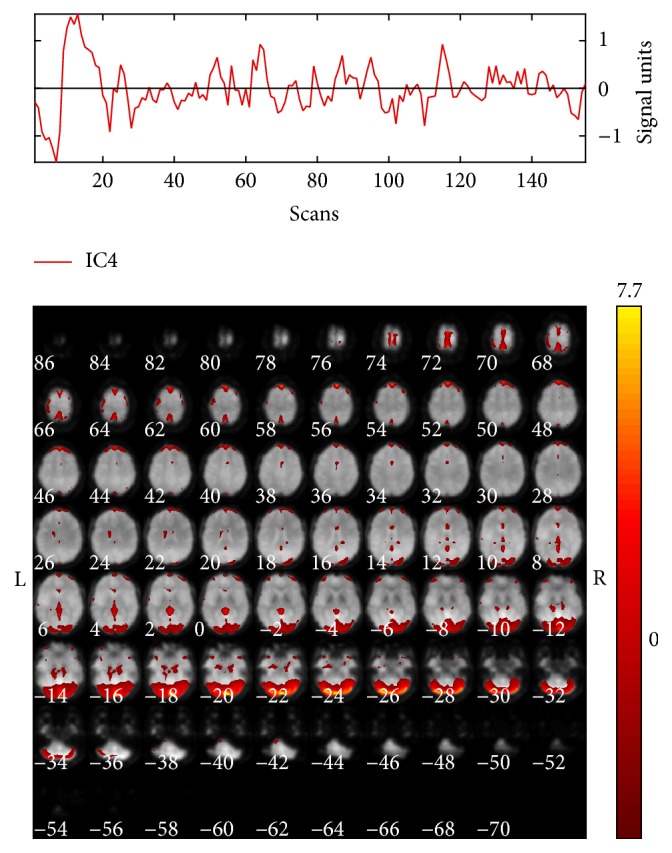
Spatial activation map and time course of IC4 “cerebellar-medial-frontal,” color bar represents *t* values.

**Figure 5:**
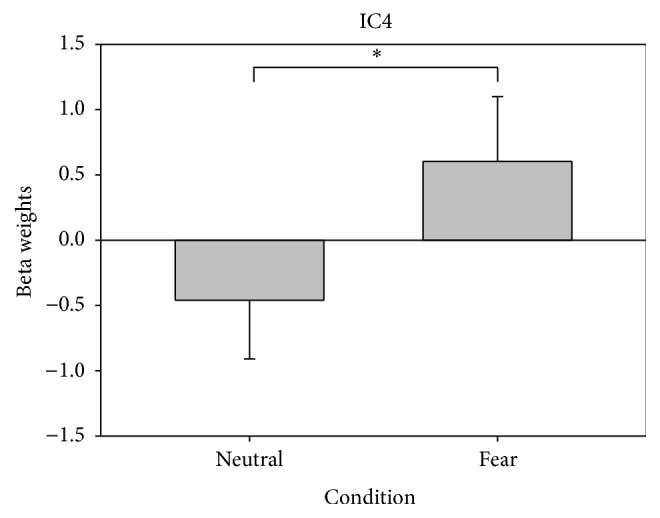
Modulation of IC4 in the neutral and fear condition of Face Matching Task. ^*∗*^ *P* < 0.05.

Since our aim was to characterize functionally connected brain networks during aversive processing and given that the only difference between the neutral and fear condition was the expressed emotion, the neutral condition represents a more subtle control condition for fear processing; therefore beta values were obtained by multiple regression of both conditions. The repeated-measures ANOVA showed a significant component related to the aversive emotional processing (IC4) (main effect of condition: *F*_1,9_ = 5.99, *P* = 0.037). This component presented a pattern of positive modulation during aversive emotional processing; the corresponding network included frontal, limbic, occipital, temporal, and cerebellar regions, so it was named “cerebellar-medial-frontal” (Table 2, Figures 4 and 5).

Results obtained from comparing neutral and fear conditions showed a positive modulation in a network predominantly cerebellar-medial-frontal, which also included activations in temporal regions. Activations in regions such as parahippocampal, frontal, and fusiform gyrus are according to the findings reported in paradigms of face emotional processing and recognition in passive view [10], shifting of the attentional focus [11], and implicit emotional tasks [12]. Specifically, our results are consistent with those reported using FMT in aversive emotional conditions (faces of fear and anger) against a motor control task (geometrical shapes) [2, 16].

It is noteworthy that studies which have used the FMT have methodological differences in the contrasts between conditions. Traditionally, reported results are based on activations to aversive emotions (fear + anger) compared with control tasks that involve the pairing of geometric shapes. One of the advantages of using the FMT is its potential value as a biomarker of disorders and behavioral traits associated with negative affectivity [14, 17, 18, 20–22]. It has been suggested that the processing of faces of fear plays an important role in social interaction, as it allows modulating the behavioral response by observing the emotional state of the other [3]. In this sense the negative affect is more related to fear: fear stimuli cause states of distress and this is perceived by the observer, facilitating socioemotional adaptive responses [47]. Therefore, we believe that adapting the FMT to a condition of fear and making a direct comparison with the neutral condition allowed us to detect the neural network that may be more associated with negative affectivity.

On the other hand, previous studies of FMT have focused on the amygdala activation, using ROIs and SVC analysis, reaching very consistent results regarding the role of the amygdala in the aversive emotional processing [14, 17–19]. In this sense, our results were not according to the differential activation of the amygdala between neutral and fear conditions; this may be due to differences in the analysis used in previous studies and the present study.

Seed studies based on ICA and FMT have yielded interesting results. Seed analysis is based on the extraction of the time course of a ROI, which is associated with the presentation of a stimulus; correlation maps are then obtained between the time courses of the remaining voxels and the time course of the ROI [30]. By using seed analysis amygdala activation in aversive emotion conditions has been reported; this activation modulated the functional connectivity in brain structures, included in the medial-frontal-occipital IC4 reported in this study [26, 30]. Therefore it is probable that the analysis used in the present study is sensitive enough to detect the modulation of other regions outside the amygdala that are involved in the aversive emotional processing. Other studies of ICA and FMT have focused on the modulation of resting state such as the DMN and frontocingulate network, concluding that the modulation reported in these networks can be a good indicator of emotional reactivity and internal emotional monitoring [28, 29]. It is interesting that the stimuli used by Cisler et al. [28] are fearful faces and that the activity of frontocingulate network has been associated with the interoceptive sensory integration, suggesting that the increase in activity in conditions of fear is associated with the development of adaptive responses.

One of the structures that showed a temporally coherent modulation during the fear condition within the IC4 was the superior temporal gyrus. This region in its anterior portions is anatomically connected with the orbitofrontal cortex and the amygdala [5, 48], and it has been reported that alterations in structural connectivity between these regions may predispose to behaviors related to alterations in emotional processing, such as violence [49]. The superior temporal gyrus, in animal and human models, has been implicated in socioemotional processing [5, 50] indicating that the anatomical and functional integrity of this structure is essential for social interaction, since it allows modulating acceptable social responses.

In fMRI studies about the processing of abstract concepts that define social behaviors, activations have been reported in anterior regions of the right superior temporal gyrus in healthy subjects [51]; this is relevant as it has been proposed that the knowledge of these social actions, together with the emotional recognition and expression, is critical to establishing and maintaining relationships [51, 52].

Other structures within the IC4, whose modulation in the fear condition was of interest, were those belonging to the cerebellum. Through ICA it was possible to detect that these regions are functionally connected with other corticosubcortical regions involved in emotional processing. The regulatory role of the cerebellum in the higher functions such as emotional processing has been previously described; in fact it has been described that cerebellar lesions produce significant clinical alterations in emotional processing and social skills [53–55]. In a recent study of functional connectivity in resting state, it was suggested that violent behavior, characterized by abnormalities in emotional processing, is related to dysfunction in a cerebellum-prefrontal cortex neural network, which was differentially connected between control and violent groups [56]. This evidence in resting state, together with the results of positive modulation and cerebellar functional connectivity within IC4 of the present study, suggests that the cerebellum is involved in emotional processing.

Another interesting finding was the negative modulation of IC2 which, as mentioned previously, includes regions involved in emotional processing.

ICA allows decomposing the observed BOLD signal into independent components; however, a limitation of this technique is the accuracy to detect which state of the cognitive process or which feature of the stimulus is associated with each time course [24]. In order to understand the type of modulation of the ICs of interest (i.e., IC4 had a positive modulation in the fear condition while IC2 presented a negative modulation to facial stimuli), the coefficients of temporal correlation between the onsets of fear and the time courses of these two components were reviewed. We observed that the correlation of the positively modulated IC4 was higher than the correlation of the negatively modulated IC2 (*r*_IC4_ = 0.1; *r*_IC2_ = 0.07), which may indicate that the activity observed in these components may be associated with different aspects of the emotional processing. That is, positively modulated IC4 could be associated with the perception and integration of emotional stimulus, while negatively modulated IC2, that was less temporally correlated with the onset of fear stimulus and included structures associated with emotional processing, may be associated with regulatory processes of emotion. In this sense it has been reported that the training of reappraisal skills decreases the response to stimuli that provoke emotional reactivity in brain regions involved in facial emotional processing [57]. In behavioral studies it has been suggested that reappraisal of emotional stimuli is present even in an implicit way; that is, a high level of emotional awareness is not necessary to reduce emotional reactivity [58]. It is likely that the sample of the present study, that was composed of healthy subjects, has an implicit mechanism of reappraisal of emotional stimuli that could be expressed as a negative modulation in active regions during processing of emotional stimuli.

As a limitation of the present study we refer to the phenomenon of reappraisal of emotional stimuli as a possible mechanism that may be related to the negative modulation of IC2. In future studies it would be interesting to make an experimental design to evaluate this phenomenon directly. One of the strengths was the modification of FMT including fearful stimuli, which are more directly associated with negative affectivity. As previously mentioned, the FMT has been considered a good biological marker of this personality trait.

## 4. Conclusions

In summary, the results of the present study allowed us to observe that there are temporally coherent neural networks whose modulation, positive or negative, contributes to a complex phenomenon such as emotional processing. Through different statistical strategies we were able to disentangle some aspects of the emotional processing and also to detect differential modulation within brain structures implicated in both facial and emotional processing.

ICA has advantages in the decomposition of the observed BOLD signal and has an important clinical value to detect functionally connected neural networks during cognitive processing. We believe that, from the perspective of brain function, ICA captures many of the theoretical principles about biological plausibility, resulting in more efficient modeling of the neural dynamics.

## Supplementary Material

In supplementary material the processes of screening and sample selection, and the results of the second-level anaylisis of the Face Matching Task paradigm are presented.

## Figures and Tables

**Table 1:** Functionally connected brain regions with a strong negative modulation within IC2 in fear condition.

IC	Area	BA	Volume (cc) left/right	Random effects: max *t* value (*x*, *y*, *z*), left	Random effects: max *t* value (*x*, *y*, *z*), right
IC2 parahippocampal-prefrontal	Parahippocampal gyrus	35, 36	0.1/0.1	6.3 (−36, −32, −20)	3.6 (24, −22, −22)
	Inferior frontal gyrus	9	0.2/0.0	5.5 (−48, 5, 29)	
	Fusiform gyrus	37	0.1/0.1	4.7 (−46, −55, −17)	3.8 (55, −49, −18)
	Anterior cingulate		0.0/0.1		4.6 (10, 17, 25)
	Subgyral	6	0.1/0.2	4.0 (−10, −44, 11)	4.5 (24, −7, 56)
	Middle temporal gyrus	21	0.1/0.1	4.3 (−40, −63, 27)	3.9 (51, −43, −13)
	Thalamus		0.0/0.1		4.1 (10, −11, 15)
	Uncus	34	0.1/0.0	3.9 (−14, −7, −22)	
	Extranuclear		0.1/0.0	3.9 (−4, −15, 14)	
	Inferior parietal lobule		0.0/0.1		3.7 (36, −24, 27)
	Precentral gyrus		0.1/0.0	3.6 (−53, 4, 35)	
	Declive		0.1/0.0	3.6 (−50, −57, −19)	

Notes: IC = independent component; BA = Brodmann area.

**Table 2:** Functionally connected brain regions with a strong positive modulation within IC4 in fear condition.

IC	Area	BA	Volume (cc) left/right	Random effects: max *t* value (*x*, *y*, *z*), left	Random effects: max *t* value (*x*, *y*, *z*), right
IC4 cerebellar-medial-frontal	Declive		3.3/2.0	9.7 (−26, −67, −22)	7.0 (30, −61, −17)
	Extranuclear		0.1/0.1	4.4 (−2, −23, 9)	6.7 (2, −23, 9)
	Culmen		0.4/0.3	6.4 (−26, −53, −19)	6.0 (8, −68, −8)
	Cuneus	17	0.2/0.3	5.8 (−24, −85, 12)	4.8 (16, −91, 3)
	Uvula		0.2/0.0	5.6 (−26, −79, −23)	
	Inferior occipital gyrus	18	0.3/0.0	5.5 (−18, −90, −9)	
	Lingual gyrus	17, 18	0.5/0.8	4.6 (−8, −86, −14)	5.4 (30, −70, −7)
	Fusiform gyrus	19, 37	0.5/0.3	4.8 (−36, −80, −14)	5.2 (26, −68, −7)
	Tuber		0.2/0.0	5.1 (−46, −61, −24)	
	Middle occipital gyrus		0.3/0.6	5.0 (−26, −89, 10)	5.0 (24, −91, 5)
	Superior frontal gyrus	6	0.0/0.1		4.8 (6, 19, 60)
	Precentral gyrus	4	0.2/0.0	4.2 (−36, −19, 56)	
	Subgyral		0.0/0.1		4.2 (44, −63, −7)
	Culmen of vermis		0.1/0.0	4.0 (−2, −66, −7)	
	Medial frontal gyrus	6	0.0/0.1		3.9 (2, 47, 42)
	Declive of vermis		0.1/0.1	3.7 (−2, −72, −10)	3.7 (4, −69, −12)
	Thalamus		0.0/0.1		3.7 (4, −19, 8)
	Pyramis		0.1/0.0	3.7 (−4, −75, −23)	
	Parahippocampal gyrus		0.1/0.0	3.6 (−16, −31, −5)	
	Superior temporal gyrus		0.0/0.1		3.6 (44, 20, −18)

Notes: IC = independent component; BA = Brodmann area.
