# Supplementary material for: Neural Modulation in Aversive Emotion Processing: An Independent Component Analysis Study
Source: Comput Math Methods Med. 2016 Aug 8;2016:2816567. doi: 10.1155/2016/2816567 (PMC4992784; doi:10.1155/2016/2816567)
Supplement: Supplementary file 1 — In supplementary material the processes of screening and sample selection, and the results of the second-level anaylisis of the Face Matching Task paradigm are presented. [file 2816567.f1.docx]

**Screening and sample selection**

To select the final sample of healthy subjects, a set of screening questionnaires was used to discard traits associated to cognitive alterations. The problem drinking of alcohol was assessed through the Alcohol Use Disorders Identification Test (AUDIT) [1]. The level of dependence to nicotine was assessed through the Spanish version of Fagerström Test for Nicotine Dependence [2]. Anxiety traits were assessed through the Spanish version of the Beck’s Anxiety Inventory (BAI) [3]. Depression traits was assessed through the Spanish version of Beck’s Depression Inventory (BDI) [4]. Aggression was assessed through the Spanish version of Reactive Proactive Aggression Questionnaire (RPQ) [5]. Borderline personality disorder traits were assessed through the Spanish version of the Borderline Symptoms List (BSL-23) [6]. Impulsivity traits were assessed through the Spanish version of Plutchik’s Impusivity Scale [7]. This screening battery was applied to a total of 217 voluntaries, 42 met the inclusion criteria about the absence of mentioned traits. Then the 42 subjects were invited to participate in the present study, 17 agreed to participate voluntarily in the 2 sessions described as follows:

*Session 1.* In this session a clinical history was carried out to explore the medical and neurological background, subjects were also assessed through the Mini-International Neuropsychiatric Interview (M. I. N. I.) [8] to discard psychiatric traits and substance abuse and dependence. Subjects with any psychiatric trait were excluded for the next sessions, one subject was excluded according to this criteria. If the subject did not had psychiatry traits, medical and/or neurological background, a neuropsychological evaluation was carried out through the “Evaluación neuropsicológica breve en español NEUROPSI” [9], this neuropsychological battery asses nine cognitive domains, offers a global score of cognitive integrity, and also offers a qualitative profile of the neuropsychological performance. The subjects were excluded if had a global score below of normative data according to age and years of education and/or presented an alteration according to qualitative profile. 3 subjects were excluded according to this criteria. This session lasted 90 minutes approximately. The 13 remaining subjects were invited to the second session.

*Session 2.* In this session the magnetic resonance images were acquired, of the 13 subjects, 2 did not attend, and the images of one subject were discarded because artifacts. Structural and functional images were acquired. The subjects practiced the experimental paradigms outside the scanner, then the subjects were scanned during the resolution of 3 cognitive tasks, in the present study the results of the Face Matching Task were reported. This session lasted 120 minutes approximately.

Once this procedure was concluded, the images of 10 healthy subjects were analyzed. The results of the screening battery and neuropsychological assessment of the final sample are presented in Table S1.

TABLE S1. Results of the screening battery and neuropsychological assessment.

|  | Mean + SD | Exclusion criteria |
| --- | --- | --- |
| AUDIT | 4 + 2.86 | > 8 |
| Fagerström test | 0.66 + 1.15 | > 3 |
| BAI | 4.5 + 2.12 | > 7 |
| BDI | 3.7 + 2.83 | > 9 |
| RPQ* | 3.2 + 2.09 | > 8 |
| BSL-23 | 4.4 + 2.2 | > 48 |
| Plutchik’s Impulsivity Scale | 13 + 4.13 | > 20 |
| NEUROPSI Global score | 120.8 + 3.08 | < 102 |

*Reactive subscale.

**GLM analysis results**

The results of the second level GLM analysis (one sample T test, P < 0.005 (uncorrected), k = 10) are presented in Table S2. Also the maps of activation in the contrasts of interest are presented in Figures S1, S2, S3.

TABLE S2. Brain regions that showed activation in contrast of interest.

| Regions^a^ | BA | Side | x y z (mm)^b^ | Z | P<0.005 (uncorr) |
| --- | --- | --- | --- | --- | --- |
| *Faces (neutral+fear > control)* |  |  |  |  |  |
| Fusiform gyrus | 37 | L | -44 -54 -14 | 4.009 | 0.00003 |
| Lingual gyrus | 18 | L | -12 -76 8 | 3.336 | 0.0004 |
| Cuneus | 17 | L | -8 -84 14 | 2.763 | 0.002 |
|  |  | R | 10 8 -18 | 3.279 | 0.0005 |
| Precuneus | 31 | L | -20 -50 42 | 3.028 | 0.001 |
| Lentiform nucleus | Putamen | L | -24 8 0 | 2.842 | 0.002 |
|  |  |  |  |  |  |
| *Fear > Neutral* |  |  |  |  |  |
| Precentral gyrus | 6 | R | 34 -4 50 | 4.182 | 0.00001 |
| Cingulate gyrus | 24 | R | 26 2 32 | 3.630 | 0.0001 |
| Precentral gyrus | 4 | R | 34 -14 42 | 3.607 | 0.0001 |
| Claustrum |  | L | -30 20 -4 | 3.852 | 0.00005 |
| Lentiform nucleus | Putamen | L | -30 8 4 | 3.211 | 0.0006 |
| Lentiform nucleus | Putamen | L | -26 16 4 | 3.153 | 0.0008 |
| Amygdala |  | R | 24 -2 -22 | 3.770 | 0.00008 |
| Amygdala |  | R | 32 0 -24 | 2.694 | 0.003 |
| Insula | 13 | R | 42 -10 14 | 3.579 | 0.0001 |
| Amygdala |  | L | -20 -4 -20 | 3.576 | 0.0001 |
| Parahippocampal gyrus | 28 | R | 24 -30 -10 | 3.529 | 0.0002 |
| Middle frontal gyrus | 9 | R | 48 16 34 | 3.510 | 0.0002 |
| Superior temporal gyrus | 22 | L | -36 -50 20 | 3.399 | 0.0003 |
| Middle temporal gyrus | 39 | L | -36 -52 28 | 2.742 | 0.003 |
| Fusiform gyrus | 37 | L | -38 -62 -12 | 3.379 | 0.0003 |
| Culmen |  | L | -44 -58 -22 | 3.156 | 0.0007 |
| Declive |  | L | -38 -76 -16 | 3.313 | 0.0004 |
| Inferior frontal gyrus | 47 | L | -38 34 -4 | 3.260 | 0.0005 |
| Precentral gyrus | 13 | L | -52 -10 10 | 3.186 | 0.0007 |
| Precuneus | 31 | R | 24 -66 24 | 3.144 | 0.0008 |
| Caudate |  | R | 26 -6 20 | 3.041 | 0.001 |
| Thalamus |  | R | 14 -8 16 | 3.016 | 0.001 |
| Precentral gyrus | 4 | R | 64 -6 24 | 2.921 | 0.001 |
| Inferior frontal gyrus | 45 | R | 56 28 14 | 2.903 | 0.001 |
| Precentral gyrus | 9 | L | -44 24 34 | 2.853 | 0.002 |
| Precentral gyrus | 4 | L | -54 -10 32 | 2.853 | 0.002 |
|  |  |  |  |  |  |
| *Fear controled by neutral faces and sensorimotor activity (fear > neutral+control)* |  |  |  |  |  |
| Amygdala |  | L | -20 -6 -20 | 4.905 | 4.6519E-07 |
| Thalamus | Lateral posterior nucleus | L | -22 -20 14 | 3.974 | 0.00003 |
| Insula | 13 | L | -36 -28 22 | 3.849 | 0.00005 |
| Middle temporal gyrus | 39 | L | -30 -58 34 | 3.815 | 0.00006 |
| Superior temporal gyrus | 22 | L | -36 -48 22 | 3.553 | 0.0001 |
| Superior temporal gyrus | 39 | L | -50 -56 34 | 3.328 | 0.0004 |
| Culmen |  | L | -42 -54 -20 | 3.776 | 0.00007 |
| Declive |  | L | -38 -60 -16 | 3.549 | 0.0001 |
| Declive |  | L | -40 -76 -16 | 3.003 | 0.001 |
| Caudate |  | R | 22 -4 20 | 3.688 | 0.0001 |
| Thalamus |  | R | 12 -6 18 | 2.861 | 0.002 |
| Postcentral gyrus | 2 | L | -36 -20 32 | 3.585 | 0.0001 |
| Thalamus |  | L | -6 -22 10 | 3.584 | 0.0001 |
| Parahippocampal gyrus | 28 | R | 24 -28 -12 | 3.526 | 0.0002 |
| Lentiform nucleus | Putamen | L | -20 4 12 | 3.343 | 0.0004 |
| Precentral gyrus | 9 | L | -44 24 32 | 3.340 | 0.0004 |
| Claustrum |  | L | -30 20 -4 | 3.330 | 0.0004 |
| Declive |  | R | 36 -66 -14 | 3.311 | 0.0004 |
| Inferior frontal gyrus | 47 | R | 40 30 -16 | 3.248 | 0.0005 |
| Inferior frontal gyrus | 47 | R | 40 36 -6 | 3.230 | 0.0006 |
| Inferior frontal gyrus | 47 | R | 30 28 -14 | 2.891 | 0.001 |
| Superior frontal gyrus | 8 | L | -8 40 44 | 3.206 | 0.0006 |
| Inferior frontal gyrus | 47 | L | -38 34 -8 | 3.191 | 0.0007 |
| Amygdala |  | R | 24 -2 -24 | 3.174 | 0.0007 |
| Declive |  | L | -12 -78 -8 | 3.146 | 0.0008 |
| Fusiform gyrus | 20 | R | 46 -42 -18 | 3.114 | 0.0009 |
| Middle frontal gyrus | 46 | R | 48 32 16 | 3.079 | 0.001 |
| Inferior frontal gyrus | 9 | R | 58 24 16 | 3.016 | 0.001 |
| Precentral gyrus | 6 | R | 62 0 20 | 3.036 | 0.001 |
| Claustrum |  | L | -30 6 14 | 2.994 | 0.001 |
| Insula | 13 | L | -46 -6 14 | 2.988 | 0.001 |
| Precentral gyrus | 6 | L | -52 -8 8 | 2.647 | 0.004 |
| Middle frontal gyrus | 9 | R | 48 18 32 | 2.976 | 0.001 |
| Cingulate gyrus | 32 | L | -12 26 30 | 2.955 | 0.001 |
| Postcentral gyrus | 3 | R | 36 -16 40 | 2.806 | 0.002 |
| Middle frontal gyrus | 6 | R | 34 -4 48 | 2.735 | 0.003 |
| Middle occipital gyrus | 18 | L | -24 -88 -6 | 2.710 | 0.003 |

Notes: ^a^Talairach labels; BA=Brodmann areas; Side= L left, R right; ^b^MNI coordinates.


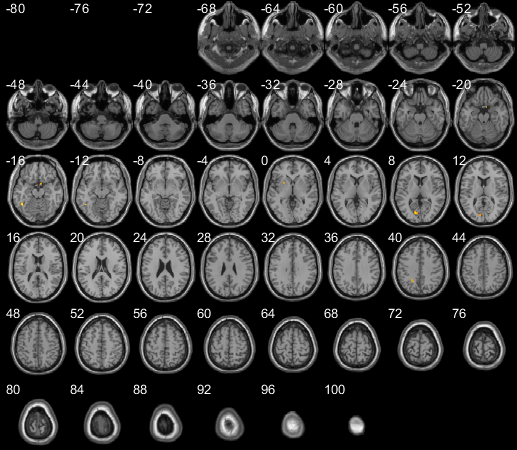


Figure S1. Axial view of faces activation (neutral+fear > control).


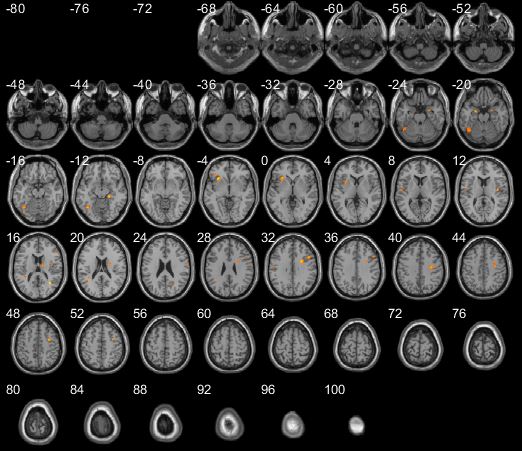


Figure S2. Axial view of fear activation (fear > neutral).


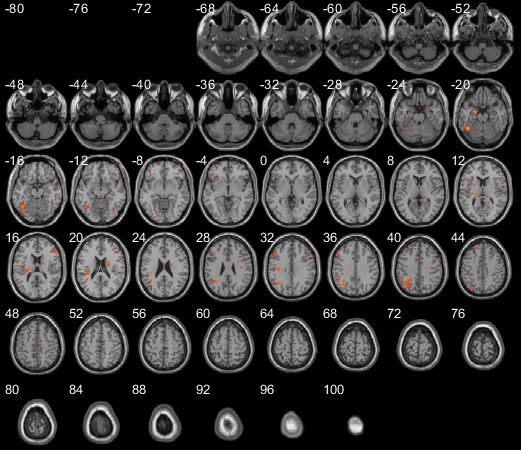


Figure S3. Axial view of fear activation controlled by neutral faces and sensorymotor activty (fear > neutral+control).

**References**

1. J. B. Saunders, O. G. Aasland, T. F. Babor, J. R. de la Fuente, and M. Grant, “Development of the Alcohol Use Disorders Identification Test (AUDIT): WHO collaborative project on early detection of persons with harmful alcohol consumption-II,” *Addiction,* vol. 88, pp. 791-804, 1993.
2. E. Becoña and F. L. Vázquez, “The Fagerström Test for Nicotine Dependence in a Spanish sample,” *Psychological Reports,* vol. 83, no. 3 Pt 2, pp. 1455-1458, 1998.
3. R. Robles, R. Varela, S. Jurado, and F. Páez, “Versión Mexicana del Inventario de Ansiedad de Beck: propiedades psicométricas,” *Revista Mexicana de Psicología*, vol. 18, no. 2; pp. 211-218, 2001.
4. S. Jurado, M. E. Villegas, L. Méndez, F. Rodríguez, V. Loperena, and R. Varela, “La estandarización del inventario de depresión de Beck para los residentes de la ciudad de México,” *Salud mental*, vol. 21, no. 3, pp. 26-31, 1998.
5. J. M. Andreu, M. E. Peña, and J. M. Ramírez, “Cuestionario de Agresión Reactiva y Proactiva: un instrumento de medida de la agresión en adolescentes. *Revista de Psicopatología y Psicología Clínica*, vol. 14, no. 1, pp. 37–49, 2009.
6. J. Soler, D. Vega, A. Feliu-Soler et al., “Validation of the Spanish versión of the borderline symptom list, short form (BSL-23),” *BMC Psychiatry*, vol. 13, no. 1, pp. 39-46, 2013.
7. F. Páez, A. Jiménez, A. López, J. Raúl, H. Ortega, and H. Nicolini, “Estudio de validez de la traducción al castellano de la Escala de Impulsividad de Plutchik,” *Salud Mental*, vol. *19*, pp. 10–12, 1996.
8. D. Sheehan, Y. Lecrubier, H. Sheehan et al., “The Mini-International Neuropsychiatric Interview (M.I.N.I): the development and validation of a structured diagnostic psychiatric interview for DSM-IV and ICD-10,” *Journal of Clinical Psychiatry*, vol. 59, no. 20, pp. 22–33, 1998.
9. F. Ostrosky, A. Ardila, and M. Rosselli, *NEUROPSI Evaluación Neurospicológica Breve en Español. Manual instructivo y protocolo de aplicación,* Bayer, D. F., México, 1997.
